# Gel electrophoresis in a polyvinylalcohol coated fused silica capillary for purity assessment of modified and secondary-structured oligo- and polyribonucleotides

**DOI:** 10.1038/srep19437

**Published:** 2016-01-18

**Authors:** Martyna Barciszewska, Agnieszka Sucha, Sandra Bałabańska, Marcin K. Chmielewski

**Affiliations:** 1 Institute of Bioorganic Chemistry, Polish Academy of Sciences, Noskowskiego 12/14, 61-704 Poznań, Poland; 2 Poznan Science and Technology Park, Adam Mickiewicz University Foundation, Rubież 46, 61-612 Poznań, Poland; 3 FutureSynthesis sp. z o.o. ul, Rubież 46, 61-612 Poznań, Poland

## Abstract

Application of a polyvinylalcohol-coated (PVA-coated) capillary in capillary gel electrophoresis (CGE) enables the selective separation of oligoribonucleotides and their modifications at high resolution. Quality assessment of shorter oligomers of small interfering RNA (siRNA) is of key importance for ribonucleic acid (RNA) technology which is increasingly being applied in medical applications. CGE is a technique of choice for calculation of chemically synthesized RNAs and their modifications which are frequently obtained as a mixture including shorter oligoribonucleotides. The use of CGE with a PVA-coated capillary to analyze siRNA mixtures presents an alternative to conventionally employed techniques. Here, we present study on identification of the length and purity of RNA mixture ingredients by using PVA-coated capillaries. Also, we demonstrate the use of PVA-coated capillaries to identify and separate phosphorylated siRNAs and secondary structures (e.g. siRNA duplexes).

A wide variety of applications in modern molecular biology manifest the growing demand for modified and unmodified oligoribonucleotides. Therefore, the high quality of these biopolymers, expressed in their content, integrity (degradation level) and purity as well as identity are of crucial importance. This aspect gets special attention when oligonucleotides are considered as potential tools in multiplex PCR^1^, cloning^2^, mutagenesis or antisense methods^3^. Factors such as short analysis time, high resolution and reproducibility, accurate quantitation as well as automation in collecting data analysis are essential in a decision making process for choosing a quality control technique. All these factors are incorporated by capillary electrophoresis techniques, which combine the advantages of high pressure liquid chromatography (HPLC) and poly acrylamide slab gel electrophoresis (PAGE) with unprecedented resolution and speed^4^,^5^,^6^.

PAGE is routinely applied for the synthetic oligonucleotide analysis or purification. Despite its indisputable advantages, such as low cost, easy operation and possibility to analyze multiple samples in the same separation^7^, numerous drawbacks might be assigned to this technique, especially in terms of practical aspects. Drawbacks of the PAGE technique as an analytical method include gel deformation under the influence of electric field, low sensitivity and poor resolution^8^, but also the necessity of applying complex labeling for detection techniques, i.e. with silver nitrate, fluorescent dyes or radioisotopes^9^.

Along with its different versions defined by the mode of separation, CGE represents a powerful technique which has proved to be sufficiently competitive to HPLC^10^,^11^. These methods have been established for a wide range of biomolecules such as peptides^12^, proteins^13^,^14^ or both single- and double-stranded nucleic acids^15^,^16^,^17^,^18^,^19^,^20^. High sensitivity and low reagent consumptions make CGE extremely attractive and useful for analytical purposes. In-capillary detection, a high resolution^4^, an accurate quantitation, a short analysis time and an automation capability^21^ ensure the leading position of CGE among electrophoretic techniques.

Although, CGE also has some disadvantages. One of them is the short capillary lifetime, resulting in reduction of analysis reproducibility over a long period of time^22^. Additionally, due to the limited quantities of material loaded into a capillary, CGE is rarely used in preparative mode^23^. Moreover, a common problem in CGE analysis is the interaction between analyzed molecules and the internal capillary layer, which results in a biased variation of electroosmotic flow (EOF) responsible for producing unreliable data. Considering the fact that such data hamper proper interpretation, using capillaries with chemically modified coatings helps to prevent or minimize the mentioned problem. Depending on the type of the internal layer, it is possible to distinguish dynamic and static capillary coating procedures. Dynamic coatings rely on adsorption, whereas permanent coatings are based on covalent binding between the new layer and the internal capillary surface.

Linear polyacrylamide^24^ and cellulose derivatives such as quaternized cellulose^25^ are used for dynamic coating purposes, whereas cross-linked polyacrylamide^26^, dextran^27^ or polyvinyl alcohol (PVA)^28^,^29^ are commonly used as permanent coatings. A two-layered (dynamic-static) variation is also formed, where the first layer is immobilized by covalent binding, while the second uses adsorbing forces. However, the most important and desirable characteristic for CGE capillaries and HPLC columns alike is stability across the widest possible range of pH. This feature is ensured by a capillary with polyvinyl alcohol as a permanent layer which is suitable for the analysis of the nucleic acids of biopolymers with a considerable number of sensitive moieties, because the PVA coating is stable from pH 2.5–9.5^30^. The neutral character of the PVA-coating causes a decrease in interactions between positively charged components in the solution and the internal capillary cover^17^. As a consequence the wall does not attract so many cations from the electrolyte solution, resulting in the reduction of EOF and its decreased influence on separation. Moreover, the stability of PVA-coated capillaries can be enhanced with additional chemical modification with glutaraldehyde^31^ or citric acid^32^.

Owing to its unique merits, CGE has become a powerful analytical tool. It is successfully used in the purity assessment of native oligonucleotides and their modifications^17^, polymerase chain reaction (PCR) product analysis^33^, sequencing, accurate sizing of restriction fragments for genetic purposes or clinical and forensic analysis^34^. Moreover, additional applications continue to be developed^7^.

Herein we present the study on an application of a PVA-coated capillary in CGE as an effective method for separating and analyzing short synthetic RNA. The choice of separation conditions (the length of the capillary, kinds of sieving media, voltages) is based on literature data^35^ or experimental research for short DNA^36^. Because there are no reports that unambiguously show the advantages of PVA-coated capillary for analysis of short synthetic RNA, we decided to show that also short RNA is successfully separated by PVA-coated capillary. Application of a PVA-coated capillary as hydrophilic nonionic coating proved to be particularly useful in separating short-chain RNA from their phosphorylated analogs as well as oligomers of mixed sequences and defining their quality and purity. Moreover, this technique enables the identification of such secondary structures as duplexes composed of complementary small interfering RNA (siRNA) strands from their primary structures.

## Results & Discussion

### The scope of a PVA-coated capillary analysis of short RNA

**Figure 1:**
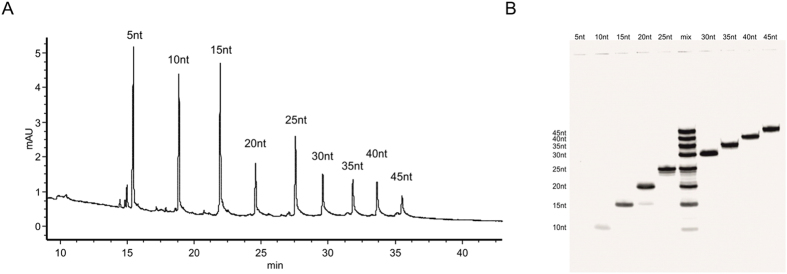
(**A**) The electropherogram shows a CGE separation of a synthetic RNA mixture. The mixture containing 9 single-stranded oligoribonucleotides (5–45 nt; length changes every 5 nt, 9 bands). CGE analysis of each sequence separately was conducted and is presented in Supplementary Information (SI). (**B**) Separation lane of a the mixture of synthetic RNA on a 15% PAGE/7 M UREA stained with SYBR Gold fluorescent dye.

Due to the correlation in the structure of DNA and RNA their migrations in sieving media are similar. We assumed that the optimal length of capillary for DNA (whose length is in the range of 5–100 nucleotides)^37^ will also be sufficient for RNA (whose length is in the range of 5–50 nucleotides). According to literature, in case of the 30 cm capillary for DNA application, run time can be lowered to 15 min, but DNA species are not well separated. Using the 60 cm capillary, the run time is about 1 h, and efficient separation is achieved. A compromise between well-resolved separation and reasonable run time could be found using 40–50 cm capillaries^30^. We applied a PVA-coated capillary of 44 cm of total length and 34 cm of effective length as an effective tool in assessing RNA quality of a synthetic RNA mixture, which is particularly visible in high resolutions, as presented in Fig. 1.

The CGE analysis is juxtaposed with the PAGE analysis of the same mixture. In order to demonstrate the separation mode of this PVA-capillary we prepared the RNA marker which is a mixture of 9 synthetic oligoribonucleotides with different lengths, where the length of oligomers increases in steps of 5 nucleotides (the shortest is 5 nt, and the longest is 45 nt). Separation of this mixture by a PVA-coated capillary will be presented by equal distribution of the oligomers on the electropherogram. Because the sequence composition of oligomers has a very close correlation with their migration time in a PVA-coated capillary, we observed significant changes of migration time for two oligoribonucleotides with the same length but different sequences. Therefore, in order to minimize the influence of the sequence on the migration time, the oligoribonucleotides mixture should be designed so that it is mainly the length of the PVA-coated capillary that has the influence on the separation of the RNA mixture components. Analysis of such a mixture should result in a separation of nucleic acids whose migration times differ by a constant distance between individual peaks. Equal distribution of the analysed mixture components on the electropherogram was realized as follows: *(i)* the sequence in shorter oligoribonucleotides (5–20 nt) was experimentally selected to give a constant distance migration between each other, *(ii)* for longer sequences (25–45 nt) of analyzed RNAs, we applied a designing method based on the fact that the shorter oligomer sequence was included in the sequence of the longer one extended with 5 additional random nucleotides. This method of sequence selection also eliminated the formation of unwanted secondary structures.

The mixture designed in such a way is characterized by high separation, which is evidently demonstrated in Fig. 1: equal distances between individual RNA mixture components are clearly shown. The equal distances also confirm that only the length of oligoribonucleotides influences the separation of the mixture components, while the influence of the sequence on migration time is almost the same for each RNA. The data presented herein explicitly proves that CGE on a PVA-coated capillary is a very useful technique for RNA quantity-quality assessment due to its high resolution.

### Purity assessment of siRNA oligonucleotides by CGE analysis

siRNAs are chemically synthesized oligonucleotides aimed at silencing individual genes in a living cell. The most commonly applied siRNAs are oligonucleotides of 18–25 nt. Their sequences are composed to complement the relevant sequence of the target gene. However, shorter oligomer sequences (post-synthetic mixture components) may be responsible for unspecific activity and translate into regulation of gene expression that differs from the intended one. Therefore, determining the presence of the shorter sequences in a sample solution is often a vital step for correct assessment of the relevant siRNA efficiency. To increase the effectiveness of siRNA activity and avoid undesirable degradation of oligoribonucleotides by using RNases, 2-deoxythymidine nucleosides are frequently introduced into oligoribonucleotides from the 3′ end. The use of PVA-coated capillaries in CGE facilitates quantitative assessment of the purity of chemically synthesized RNAs, which translates into their utility in biological applications.

**Figure 2:**
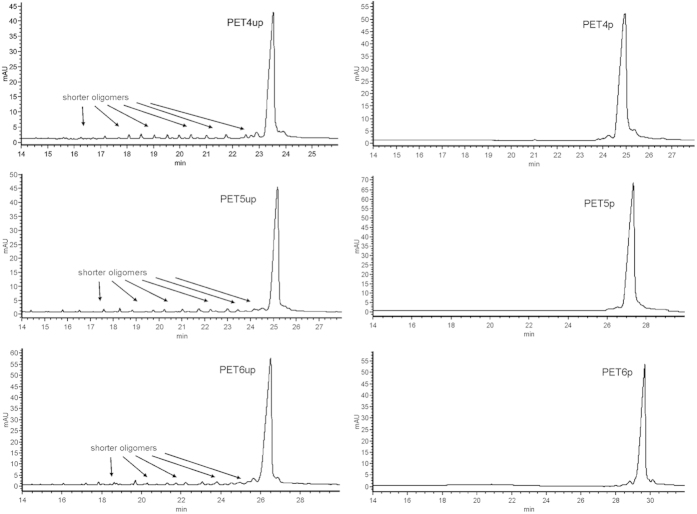
Summary of the separation of hybrid nucleic acid: RNA including 2-deoxythymidine nucleosides (21–23 nt) (**Left**) – before (“up” – unpurified) and (**Right**) – after (“p” – purified) PAGE purification.

The electropherogram (EPG) presented in Fig. 2 shows that after initial purification and desalination the chemically synthesized siRNAs are mixtures that contain the main target product as well as shorter oligoribonucleotides. Based on this analysis, it was demonstrated that it is possible using CGE to calculate the number of shorter oligomers with high resolution and the amount of the target RNA/DNA hybrid in a mixture. The results are presented in Table 1.

**Figure 3:**
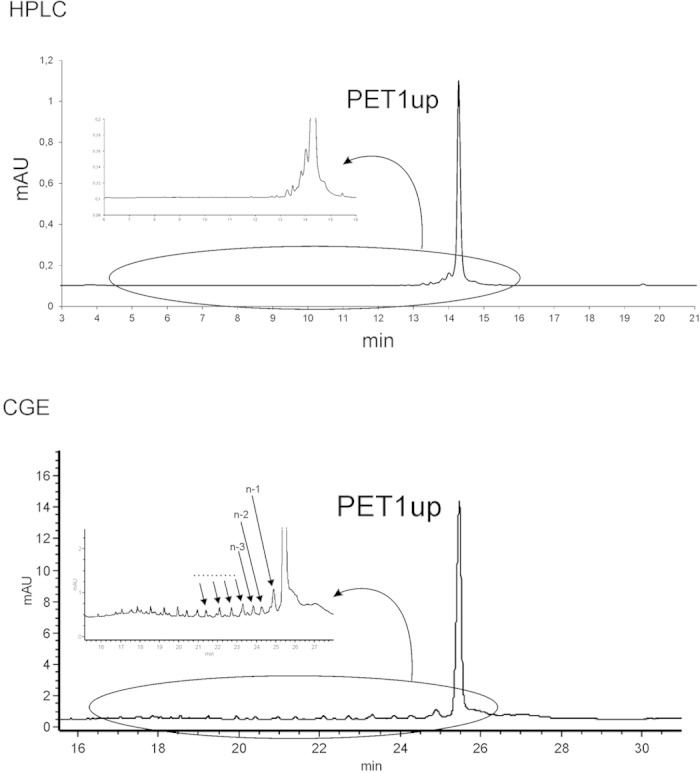
Electropherograms show a comparison of two independent separation methods: upper – HPLC and lower – CGE with a PVA-coated capillary. The circled fragment has been magnified to show the distribution of shorter oligomers.

This reveals that oligomer purification by using PAGE results in obtaining material of increased purity. The final degree of purity may be assessed more precisely via capillary electrophoresis using a PVA-coated capillary. This capillary clearly shows the effectiveness of the purification by comparing the quality of the purified and unpurified oligoribonucleotides. Purity assessment using PVA-coated capillary electrophoresis is more precise than standard HPLC on a Reversed-Phase C18 column. HPLC analysis briefly informs of the presence of a shorter nucleic acid, but it is not possible to calculate its exact quantity (see Fig. 3). Owing to the low level of separation based on HPLC, it is difficult to estimate the number and length of shorter oligoribonucleotides. However, using advanced techniques such as Fast UltraPerformance Liquid Chromatography (UPLC), allows one to obtain the same level of resolution as presented in Fig. 3 38.

The same analysis appears completely different using the PVA-coated capillary. It allows one to separate the mixture more precisely and ascribe individual signals to a oligonucleotide of a concrete length. It also enables one to calculate the number of shorter sequences coming with reduced yield at individual stages of chemical synthesis on solid support. The main oligoribonucleotides were positively verified by the MALDI TOF mass analysis where we also mainly observed the mass of the target product and the masses of shorter oligomers [the mass analysis was included in Supplementary Material].

The example of analyzed siRNAs clearly shows that CGE with a PVA-coated capillary is a useful tool for assessing the purity of nucleic acid with a mixed DNA/RNA sequence. Thanks to this technique, it is possible to calculate the number of shorter oligoribonucleotides in a mixture and assess RNA utility in molecular technologies. The subsequent section of this paper presents its application in analyses of other RNA forms and structures.

We decided to expand our study of synthetic RNAs to focus on the possibility of analyzing other structural changes. A good example to demonstrate the usefulness of PVA-coated capillary in RNA separation is the analysis of their phosphorylated forms.

### Phosphorylated oligoribonucleotides

Monoesters of phosphoric acid are important and canonic modifications in synthetic oligonucleotides applied in molecular biology. Chemical synthesis of nucleic acids enables the synthesis of such oligoribonucleotides both with and without a phosphate moiety on the 5′ or 3′ end. The process of generating phosphorylated ends in oligonucleotides is routinely used in restrictive analysis with specific endonucleases. Moreover, enzymatic methods which are applied in phosphorylation most frequently use kinases to transfer a phosphate group from donor molecules, such as ATP, to unphosphorylated oligonucleotides. Neither enzymatic nor chemical methods allow one to perform phosphorylation with 100% yield. Lower yields in phosphorylation translate in the presence of phosphorylated and unphosphorylated oligonucleotides in a mixture. Therefore, it is vital to identify whether the given oligomer boasts a phosphate moiety or not, and whether the phosphorylation/dephosphorylation process has run productively. Unfortunately, such techniques as RP-C18 HPLC or PAGE do not have sufficient resolution to show such a subtle difference between phosphorylated or unphosphorylated oligoribonucleotides. It is possible to achieve that by ion-exchange HPLC using a column suitable for oligonucleotides. However, CGE analysis on a PVA-coated capillary is also a technique that allows one to successfully separate a chemically phosphorylated ribonucleotide from its unphosphorylated analog.

**Figure 4:**
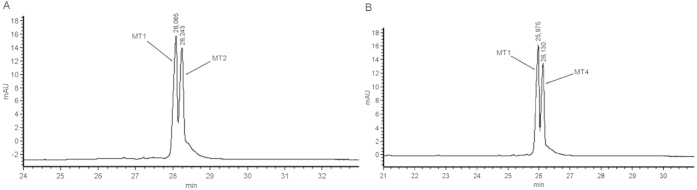
Separation of non-phosphorylated from phosphorylated short RNA (**a**) mono-5′-phosphorylated (**b**) di-3′,5′-phosphorylated. Additional information described in SI.

The two EPGs presented in Fig. 4 show separation of an oligonucleotide mixture composed of a non-phosphorylated oligoribonucleotide sequence (MT1) and analogical RNA with one or two phosphate groups (MT2 and MT4). The difference between the applied phosphorylated RNAs is the presence of an additional phosphate group on the 5′ end in the former, and 5′ and 3′ end in the latter. Increased concentration of the phosphorylated RNA caused an increase in one signal on the plot. On this basis, we ascribe the higher signal as the one coming from the phosphorylated oligonucleotide. The difference in migration time on the capillary between the phosphorylated and unphosphorylated RNA was precisely registered on the electropherogram, which allows one to calculate the contents of individual RNAs in a mixture. It is practically impossible to distinguish this structural detail via PAGE or HPLC techniques. Only CGE proves that we are dealing with separation of phosphorylation on oligoribonucleotides. Unfortunately, the separation between 3′-phosphorylated and 5′-phosphorylated RNA as well as monophosphorylated and diphosphorylated RNA in the studied conditions was not successfull (The electropherogram mixture of separation mono and di-phosphorylated short RNA was provided by SI, page 2).

**Figure 5:**
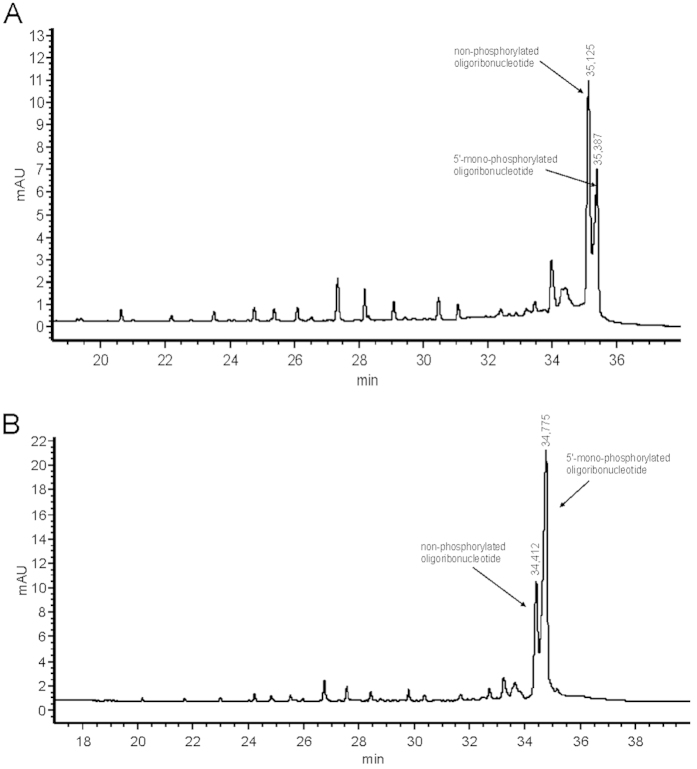
Incomplete phosphorylation of oligoribonucleotide. (**A**) electropherogram of a post-synthetic mixture of oligoribonucleotides with incomplete phosphorylation. (**B**) EPG of the same mixture with added inner standard. Signals are identified on the basis of a comparison with the inner standard.

Application of this method may be very useful for assessing the level of chemical RNA phosphorylation in the synthesis of these molecules on solid support. An analysis of the post-synthetic mixture of oligoribonucleotide with incomplete 5′-phosphorylation is depicted in Fig. 5A. In this example, the phosphorylation yield was intentionally lowered to demonstrate the usefulness of the method for identifying the phosphorylation level by using CGE on the PVA-coated capillary. In this case, we also applied a comparative analysis with an inner standard, i.e. mono-5′-phosphorylated oligoribonucleotide with an analogical sequence in order to properly identify oligoribonucleotide signals. The result is shown in Fig. 5B. As expected, the phosphorylated oligoribonucleotide exhibits longer migration time in the PVA-coated capillary. The value may be calculated owing to the high resolution of the analysis. Currently, there is no good method for assessing phosphorylation on the 5′-end of synthesized oligomers on solid support, because neither HPLC reverse phase (RP-C18) nor PAGE analyses provide such a resolution. PVA-coated capillary CGE fills this gap providing a precise tool to calculate the content of the phosphorylated ends of oligomers in the mixture.

### Secondary structure analysis of oligoribonucleotides

The gene silencing technique frequently requires using an oligoribonucleotide in the form of a double-stranded hybrid. Preparation of a double-stranded fragment consists of designing two complementary sequences, defining thermodynamic parameters and carrying out hybridization, i.e. connecting complementary strands of the future duplex. Each duplex is characterized by a defined melting temperature in which one half of the RNA assumes a single-stranded form. In standard methodology, the value is calculated theoretically and then confirmed experimentally. This approach is based on the change of UV light absorption depending on the involvement of heterocyclic bases in a secondary structure. One common method aimed at confirming the presence of a double-stranded form is based on the differences in molecule migration in gel as a result of applying voltage. The differences in migration between single- and double-stranded RNAs (ssRNA and dsRNA) are a consequence of two different separation mechanisms^39^. The “classical” sieving theory applied in electrophoresis is the so-called Ogston model and for small molecules, e.g. short RNAs, this generally explained the separation through gel pores. However, dsRNAs which are too large to be separated by sieving are separated by electrohydrodynamic forces and the separation is characterized by an inversely proportional dependence of the mobility on size and the presence of a high electric field. Those RNAs which are flexible and easily form secondary structures migrate more effectively through gel pores, as illustrated below.

**Figure 6:**
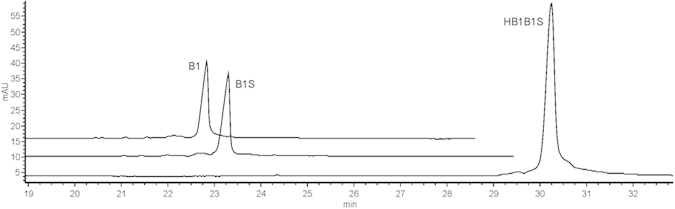
A set of electropherograms of two hybrid oligoribonucleotides (B1, B1S), containing four nucleotides with 2′-OMe modification at the 3′-end, and their duplex form (HB1 B1S). The sequences of hybrid oligoRNA were partially complementary to each other, so that they formed a secondary structure under particular conditions.

**Figure 7:**
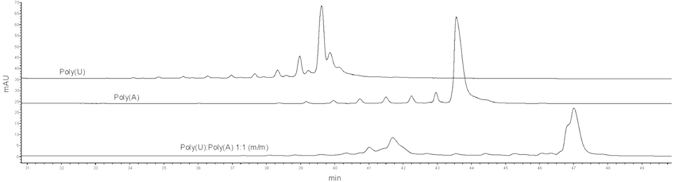
A set of electropherograms shows an analysis of sequentially homogenous oligonucleotides (30 nt): oligouridine (Poly(U)) and oligoadenosine (Poly(rA)) and their 1:1 (molar ratio) mixture.

On the basis of our experiments, we have proved that CGE with a PVA-coated capillary is an attractive technique under nondenaturing and native conditions for identifying such secondary structures as duplexes. Secondary structure differences (conjugation efficiency and duplex content) or the separation of double-stranded DNA such as restriction fragments can also be determined using native CGE^40^. The duplexes analyzed by PVA-coated capillary, whose electropherograms are presented in Figs 6 and 7, differ in terms of their considerably longer migration time from their single-stranded chains, and their identification is not problematic.

An example of a duplex which easily forms a secondary structure is RNA based on two complementary oligoribonucleotides whose sequences contain the 2′-OMe modification. This modification is responsible for increasing the stability of duplexes and meets the principles of siRNA design. An equimolar mixture of two such complementary oligoribonucleotides forms a duplex that is stable in electrophoresis conditions and is presented on electropherograms as a homogeneous signal marked by long retention time. The melting point for this duplex was theoretically calculated at 47.7 °C. The high value of the melting point guarantees that the duplex form is dominant during CGE analysis.

The second analyzed RNA duplex, which is less flexible, is formed from homogeneous polytrack sequences, and its analysis is presented in Fig. 7. In this case, the melting point of the duplex is much higher than that of the first one and theoretical calculations indicate a value of 87.3 °C. However, the CGE analysis proves a decrease in the duplex stability. The duplex is characterized by a wide peak with a shoulder as presented on electropherograms, which proves that it is not homogeneous. This results from the fact that this less flexible structure (formed from homogeneous sequences) is more steric than the pores available in gel. In addition, other structural forms are also observed, whose retention times differ from those of single-stranded forms.

Based on these results, we can also conclude that migration of RNA in a PVA-coated capillary at a constant applied voltage is also dependent on the sequence of these oligomers. This is confirmed by a significant difference in migration time between the Poly(rA) from Poly(U).

## Materials & Methods

### CE Instrumentation

Capillary Electrophoresis (CE) analyses were performed on a Capel 105M System equipped with a UV-detector (Lumex Ltd., St. Petersburg, Russia). The wavelength range for the detector was 190–380 nm with a step size of 1 nm. A polyvinylalcohol (PVA)-coated capillary (G160U-60419, Agilent, Germany) with total (L_tot_) and effective (L_eff_) lengths of 44 and 34 cm, respectively, and 100 μm ID was used for the experiments. Elforun software version 3.2.2 (Lumex Ltd., St. Petersburg, Russia) was used for system control, data collection and processing.

### Preparation of oligoribonucleotides

Oligoribonucleotides were prepared on solid support using an automatic synthesizer (H-4 model, K&A Laborgeraete GbR, Schaafheim, Germany) with the phosphoramidite approach, on a 0.2 μmole scale. A CPG support (Biosearch Technologies, Petaluma, CA, USA) appropriate to each particular oligoribonucleotide sequence and standard 3′-phosphoramidites of ribonucleosides (Glen Research, Sterling, VA, USA) were used.

All prepared and analyzed oligoribonucleotides and their sequences are shown in Table 2.

The CPG support was removed from the column and rinsed with 1 ml of AMA mixture, i.e. methylamine in water (MEA_H20_) and ammonia solution (NH_4_OH) at a ratio of 1:1 v/v for 20 min at room temperature (RT), and next heated to 65 °C for 10 min. Thereafter, the sample was evaporated to dryness and dissolved in a mixture composed of 1-methyl-2-pyrrolidinone (Sigma Aldrich, Germany), triethylamine (Sigma Aldrich, Germany) and triethylamine trihydrofluoride (Sigma Aldrich, Germany) and incubated with shaking at 700 rpm for 90 min at 65 °C. After cooling to RT, each sample was treated with 1-butanol (ROTH, Karlsruhe, Germany), incubated at −80 °C for 60 min and then centrifuged with the following parameters: 14,000 rpm, 30 min, 0 °C. The supernatant was discarded while the precipitate collected at the bottom of the tube was dried at 37 °C for 30 min. Oligoribonucleotides were purified by polyacrylamide electrophoresis under denaturing conditions with 7M urea as a denaturing agent. Two-step elution was conducted in miliQ sterile water (Merck Millipore, Darmstadt, Germany), and its efficiency was assessed directly by concentration measurements on a UV-VIS spectrophotometer BioSpec-nano (Shimadzu, Japan). After each sample was desalted on a Sephadex G-25 Column (GE Healthcare, UK), the content of oligoribonucleotide was measured using the same method.

A mixture of short RNA (length from 5–45 nucleotides) was prepared by an intermixture of equimolar amounts of the individual oligoribonucleotides dissolved in miliQ water (Merck Millipore).

Additionally, the mixture of short RNA was investigated on 15% PAGE with 7 M urea as a denaturing agent. The sample was separated under the following conditions: 450 V, 40 mA, 120 min and afterwards stained with SYBR^®^ Gold Nucleic Acid Gel Stain (Invitrogen). Detection was conducted with a laser excitation wavelength at 473 nm and an emission filter –510 nm.

### CGE chemicals

The electrophoretic oligonucleotide buffer (200 mM Bis-Tris, pH 7.2) and polymeric solution (27 (w/v)% polyethylene glycol (PEG) 35000/200 mM Bis-Tris with 20 (w/v)% acetonitrile) were prepared according to the Agilent Protocol^37^.

All buffers were passed through 0.22 μm syringe membrane filters (Millipore, Darmstadt, Germany). Directly before the analysis, the buffers were warmed up to room temperature (RT) and centrifuged with the following parameters: 12,600 rpm, 10 min, RT.

### Procedure for capillary pretreatment and analysis

Prior to the CGE analysis, oligonucleotide concentration was adjusted to 30–50 μg/ml per sample. Afterwards, the sample was mixed precisely on a vortex and centrifuged at 12,600 rpm for 10 min at RT.

The measurement was performed at the wavelength of 260 nm. CGE analyses were conducted in reversed polarity mode, with the cooling system thermostated at 30 °C.

Before every single CGE analysis, the capillary was rinsed with sterile miliQ water at 1000 mbar of applied pressure for 12 min and afterwards filled with a polymeric solution for 7 min at 2000 mbar. Each sample was injected electrokinetically under a high voltage of −10 kV for 7 s and separated at −25 kV for the time appropriate to the oligoribonucleotide length. The applied electric field during the analysis was 568.2 V/cm.

Fresh portions of the separation buffer and the oligonucleotide buffer were used in each subsequent run.

### HPLC analysis

An HPLC analysis was performed using a Shimadzu Prominence UFLC system with an LC-20AD pump and a 3 μm Luna C(18)2 100A column (15 cm × 4.6 mm) according to the following conditions: starting from 0.01 M triethylammonium acetate pH = 7.0, a linear gradient of 1% MeCN/min was pumped at a flow rate of 1 ml/min for 40 min and then held isocratically for 10 min. Peak heights were normalized to the highest peak, which was set to 1 arbitrary unit.

## Conclusions

To summarize, this article presents the results of our experimental studies showing the usefulness of the capillary gel electrophoresis technique using a PVA-coated capillary for qualitative and quantitative analysis of chemically synthesized oligoribonucleotides. This technique can distinguish between different chemically modified RNAs, quantify individual shorter RNAs in a mixture and confirm previous formation of secondary structures. The article presents the usefulness of this technique in analyzing phosphorylated oligonucleotides, particularly the RNA series, which are being increasingly applied in advanced techniques in molecular biology. Purity assessment is a decisive factor in the proper functioning of phosphorylated oligonucleotides. The results show that RNA migration is primarily influenced by its chain length, and thus the number of negative charges and nucleotide composition of the molecule.

## Additional Information

**How to cite this article**: Barciszewska, M. *et al*. Gel electrophoresis in a polyvinylalcohol coated fused silica capillary for purity assessment of modified and secondary-structured oligo- and polyribonucleotides. *Sci. Rep.* **6**, 19437; doi: 10.1038/srep19437 (2016).

## Supplementary Material

Supplementary Information

## Figures and Tables

**Table 1:** The percentage of content for a particular siRNA sequence in a mixture.

	PET 4up	PET 4p (21-mer)	PET 5up	PET 5p (23-mer)	PET 6up	PET 6p (23-mer)
Shorter oligonucleotides	17,3%	7,57%	12,14%	0,9%	12,09%	5,27%
Main oligonucleotide	82,7%	92,43%	87,86%	99,1%	87,91%	94,73%

The calculation based on the CGE analysis is presented in Fig. 2.

**Table 2:** Composition of short synthetic RNA oligonucleotides separated with CGE.

No.	Sample	Length [nt]	Sequence 5′-3′	Modification (if present)	Method of purification
1.	NORM5	5	ACCGU	—	PAGE/7M UREA; Sephadex G-25
2.	NORM10	10	ACGUGGACUU	—	PAGE/7M UREA; Sephadex G-25
3.	NORM15	15	ACGUGUCUGACUUUA	—	PAGE/7M UREA; Sephadex G-25
4.	NORM20	20	CCACGUGCUCGGAACUUACU	—	PAGE/7M UREA; Sephadex G-25
5.	NORM25	25	ACGUGACGUGUCUGAGACUUCUUUA	—	PAGE/7M UREA; Sephadex G-25
6.	NORM30	30	ACGUGACGUGUCUGAGACUUCUUUAGACUU	—	PAGE/7M UREA; Sephadex G-25
7.	NORM35	35	ACGUGACGUGUCUGAGACUUCUUUAGACUUCUUUA	—	PAGE/7M UREA; Sephadex G-25
8.	NORM40	40	ACGUGACGUGUCUGAGACUUCUUUAGACUUCUUUAUUACU	—	PAGE/7M UREA; Sephadex G-25
9.	PET1	23	AGCAGAGUUCAAAAGCCCUUCTT	dTdT-3′	PAGE/7M UREA; Sephadex G-25
10.	PET2	23	GGAGGGCUUUUGAACUCUGCUTT	dTdT-3′	PAGE/7M UREA; Sephadex G-25
11.	PET3	21	GGACAAAUCCCUUAGUCAATT	dTdT-3′	PAGE/7M UREA; Sephadex G-25
12.	PET4	21	UUGACUAAGGGAUUUGUCCTT	dTdT-3′	PAGE/7M UREA; Sephadex G-25
13.	PET5	23	GAAGGGCUUUUGAACUCUGCUTT	dTdT-3′	PAGE/7M UREA; Sephadex G-25
14.	PET6	23	AGCAGAGUUCAAAAGCCCUUCTT	dTdT-3′	PAGE/7M UREA; Sephadex G-25
15.	MT1	20	GCAGCACCAUUAAGAUUCAC	—	PAGE/7M UREA; Sephadex G-25
16.	MT2	20	P-GCAGCACCAUUAAGAUUCAC	5′-P	PAGE/7M UREA; Sephadex G-25
17.	MT4	20	P-GCAGCACCAUUAAGAUUCAC-P	5′-P and 3′-P	PAGE/7M UREA; Sephadex G-25
18.	B1	21	AAUUCGGUUGUGAACAUmCmCmCmG	2′-OMe	PAGE/7M UREA
19.	B1S	21	GGAUGUUCACAACCGAAmUmUmUmU	2′-OMe	PAGE/7 M UREA
20.	Poly (rA)	30	AAAAAAAAAAAAAAAAAAAAAAAAAAAAAA	—	Sephadex G-25
21.	Poly (U)	30	UUUUUUUUUUUUUUUUUUUUUUUUUUUUUU	—	Sephadex G-25
