# Supplementary material for: Gel electrophoresis in a polyvinylalcohol coated fused silica capillary for purity assessment of modified and secondary-structured oligo- and polyribonucleotides
Source: Sci Rep. 2016 Jan 18;6:19437. doi: 10.1038/srep19437 (PMC4726012; doi:10.1038/srep19437)
Supplement: Supplementary Information [file srep19437-s1.pdf]

Supporting Information

„ Gel electrophoresis in a polyvinylalcohol coated fused silica capillary for purity assessment of modified and secondary-structured oligo- and polyribonucleotides"

Martyna Barciszewska[1], Agnieszka Sucha[2], Sandra Bałabańska[1], Marcin K. Chmielewski[1]*

[1] *Institute of Bioorganic Chemistry, Polish Academy of Sciences, Noskowskiego 12/14, 61-704 Poznań, Poland*
[2] *Poznań Science and Technology Park, A. Mickiewicz University Foundation, Rubież 46, 61-612 Poznań,*

Content:

1. Analysis of short synthetic RNA (length 5 to 45)
2. Analysis of separation of phosphorylated and non-phosphorylated RNA
3. MS analysis of oligoribonucleotides PET and Comparison of purity assessment with CE and HPLC techniques PET1-3

1. CE analysis of short synthetic RNA using PVA-coated capillary

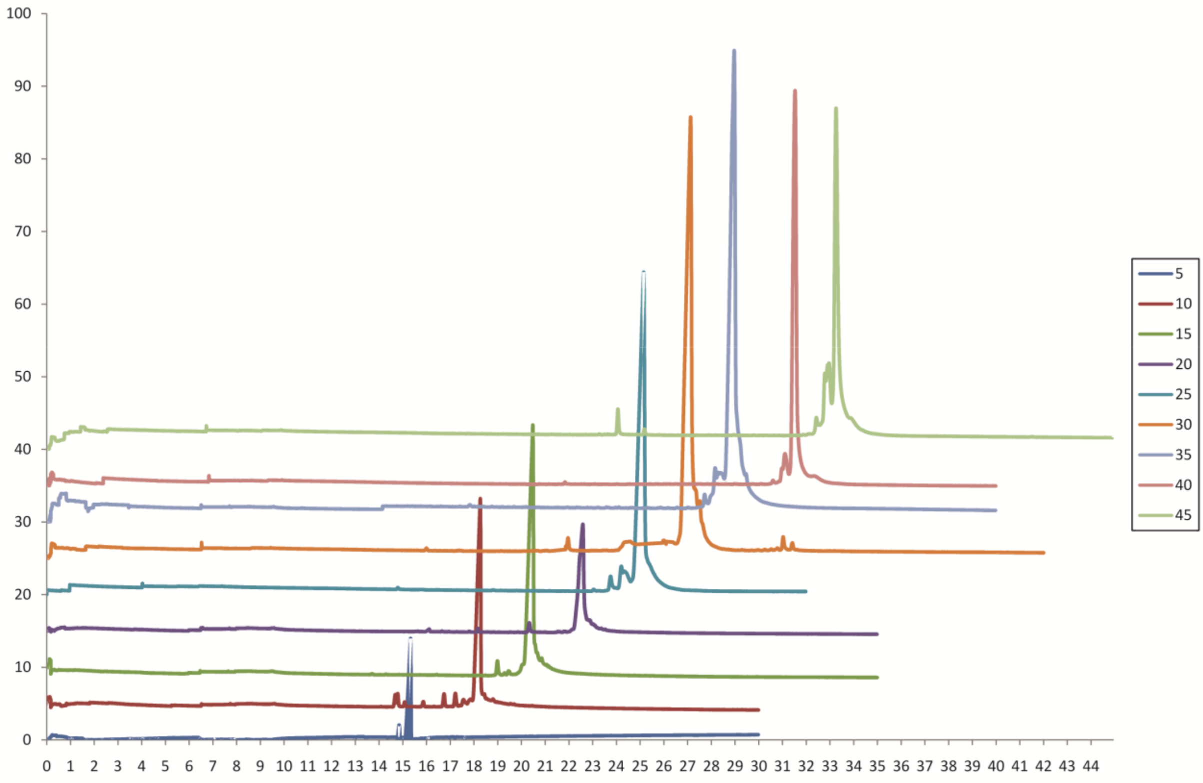

2. Analysis of separation of phosphorylated and non-phosphorylated RNA

The electropherograms presented below show separations of oligonucleotides mixtures composed of a native sequence and the analogical oligonucleotides with one (the left EPG) or two (the right EPG) phosphate groups. Differences in peak heights result from the intentionally increased concentration of the phosphorylated form of oligoribonucleotide. On the basis it was possible to properly ascribe signals to the particular sequence when the content of each sequence in the mixtures was comparable.

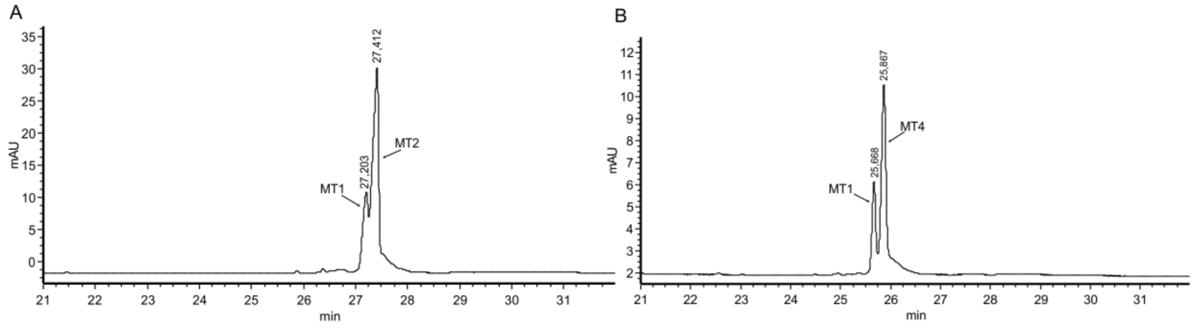

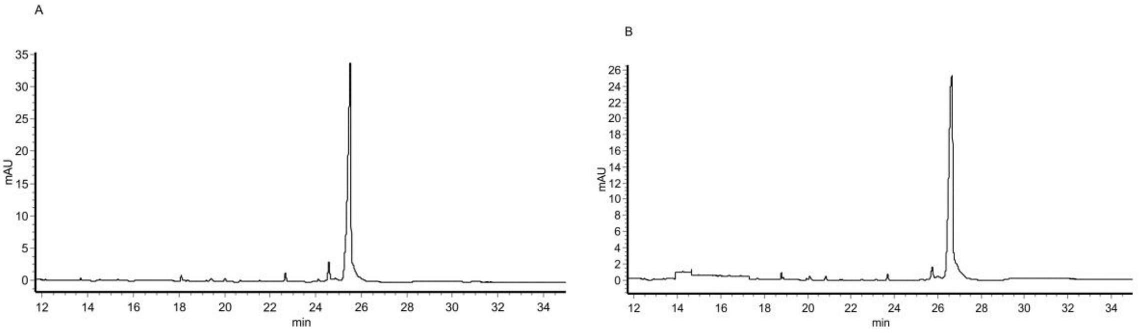

The left EPG including mixture of MT2+MT4 (1:3:2); the right EPG including MT2+MT3+MT4; (1:3:2:4)

3. MS analysis of oligoribonucleotides PET

PET4

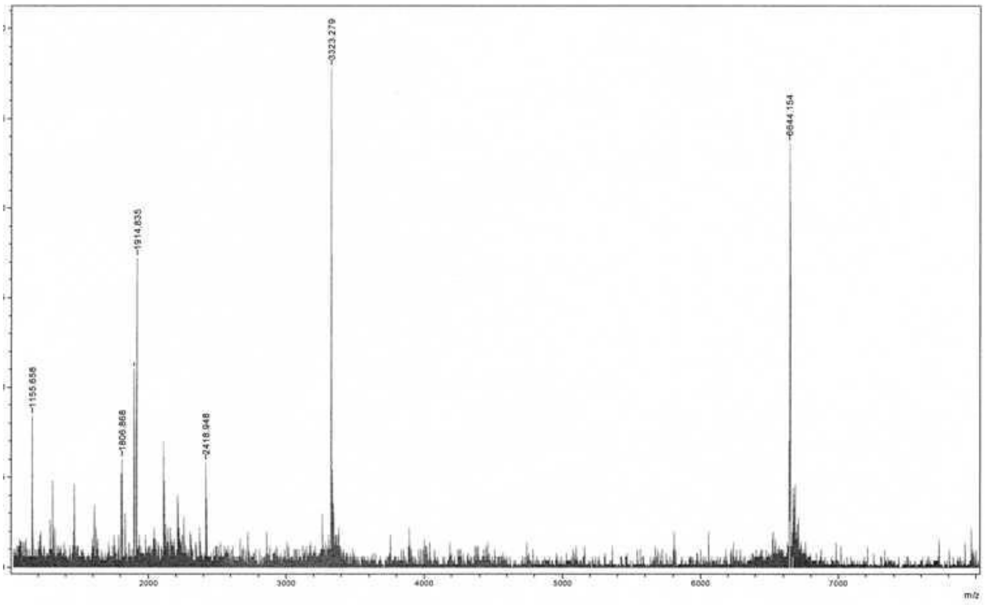

Date of acquisition	2012-04-17 13:52:21
Comment 3	
Instrument type	autoflex
Serial instrument number	25001.00217
PIE delay in [ns]	200 nsec
Voltage polarity	NEG
Comment 1	
Comment 2	
Laser repetition rate in Hz	20 psec
Linear detector voltage	1.704
Ion source voltage 1	19
Ion source voltage 2	16.9
Ion source lens voltage	8
Mass calibration Constant 1	730860.361459333
Mass calibration Constant 2	158.6871246880619
Third calib constant	-0.002349947721520408
Number of shots	30

m/z	Res.
1155.658	3760
1806.868	6105
1896.849	7107
1914.835	5635
2418.948	9551
3323.279	2509
6644.154	9220

PET5

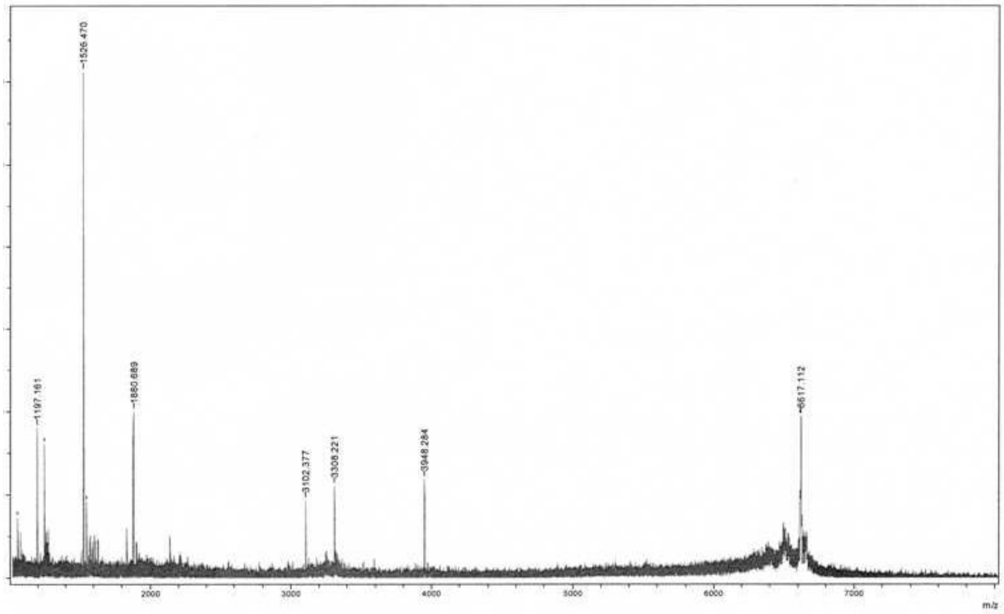

Date of acquisition	2012-04-17 13:55:40
Comment 3	
Instrument type	autoflex
Serial instrument number	25001.00217
PIE delay in [ns]	200 nsec
Voltage polarity	NEG
Comment 1	
Comment 2	
Laser repetition rate in Hz	20 psec
Linear detector voltage	1.704
Ion source voltage 1	19
Ion source voltage 2	16.9
Ion source lens voltage	8
Mass calibration Constant 1	730860.361459333
Mass calibration Constant 2	158.6871246880619
Third calib constant	-0.002349947721520408
Number of shots	30

m/z	Res.
1054.957	3338
1197.161	2854
1246.147	2088
1526.470	2497
1548.426	2609
1880.689	2284
3102.377	2206
3308.221	1747
3948.284	1363
6613.663	5955
6617.112	6684
6621.549	8004

PET6

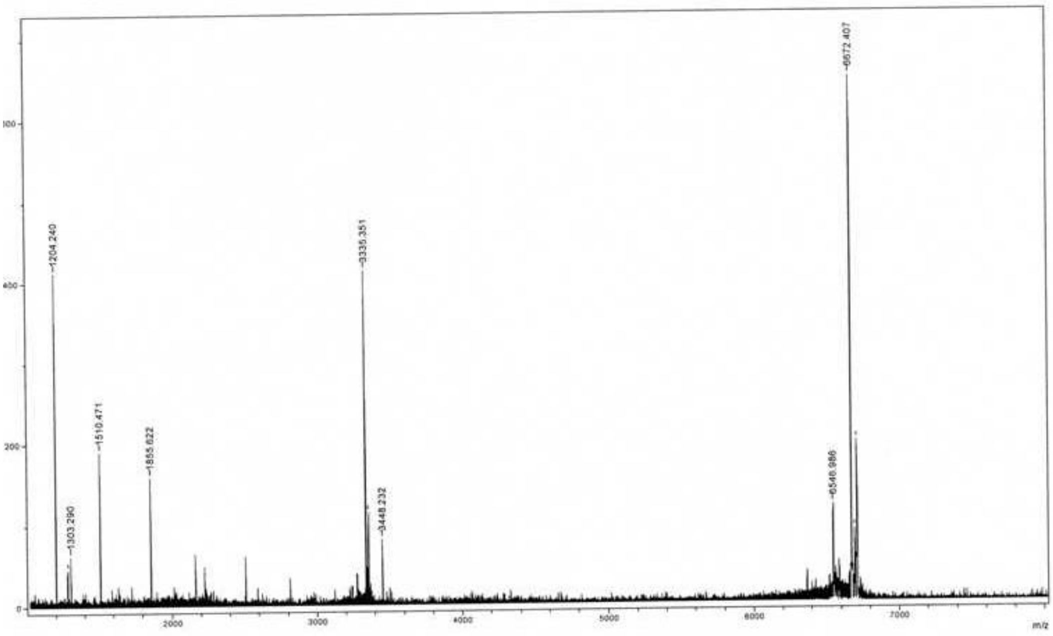

Date of acquisition	2012-04-17 13:58:28
Comment 3	
Instrument type	autoflex
Serial instrument number	25001.00217
PIE delay in [ns]	200 nsec
Voltage polarity	NEG
Comment 1	
Comment 2	
Laser repetition rate in Hz	20 psec
Linear detector voltage	1.704
Ion source voltage 1	19
Ion source voltage 2	16.9
Ion source lens voltage	8
Mass calibration Constant 1	730860.361459333
Mass calibration Constant 2	158.6871246880619
Third calib constant	-0.002349947721520408
Number of shots	30

m/z	Res.
1204.240	2617
1285.144	3758
1303.290	2810
1510.471	3300
1855.622	2882
3335.351	3349
3354.690	955
3448.232	5827
6546.986	5649
6669.128	1403
6672.407	1244
6695.394	1344
6707.933	6725

Comparison of purity assessment with CE and HPLC techniques PET1-3

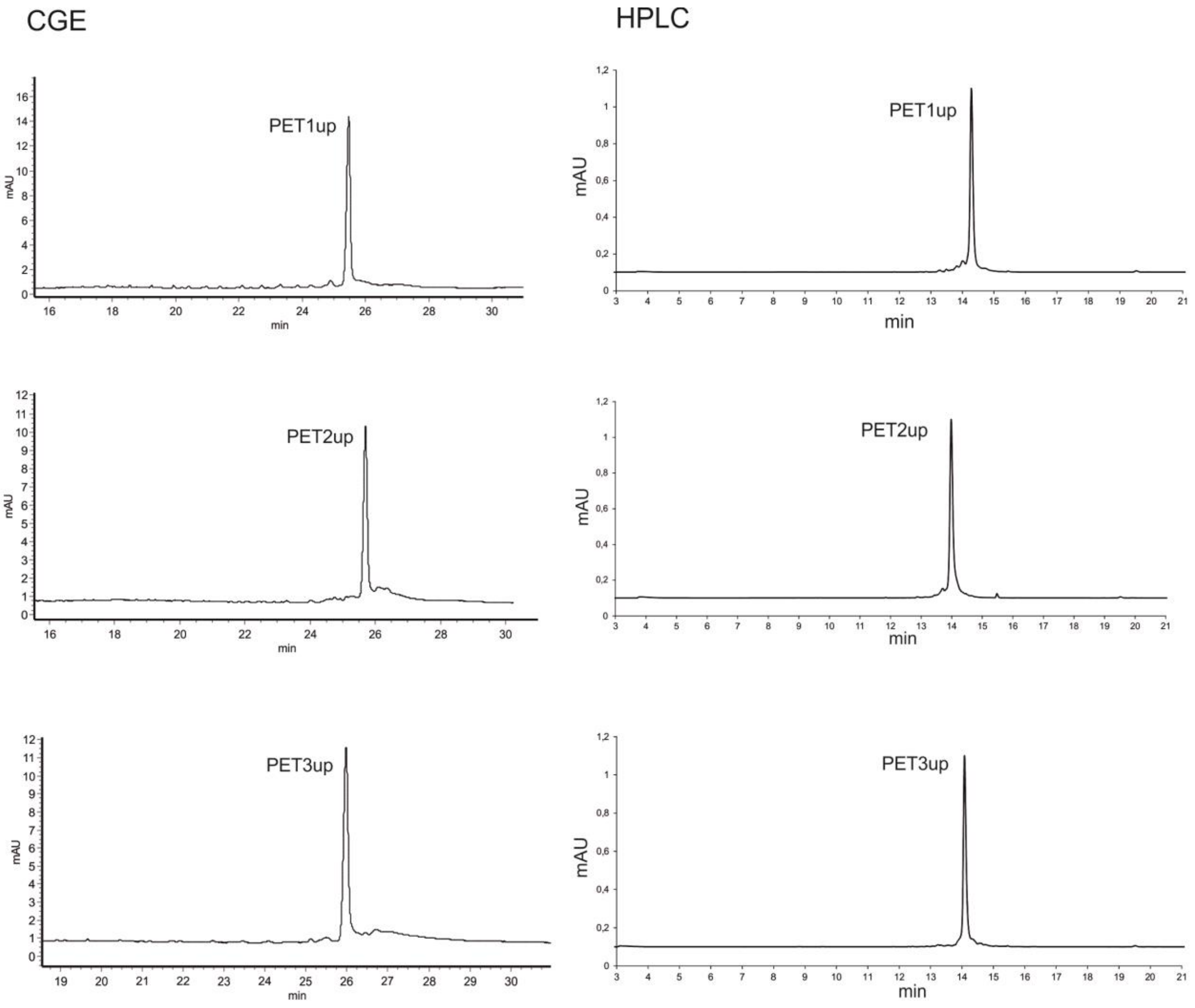
CGE
HPLC
PET1up
PET2up
PET3up
mAU
min
